# Engineering diabetic human skin equivalent for *in vitro* and *in vivo* applications

**DOI:** 10.3389/fbioe.2022.989888

**Published:** 2022-09-30

**Authors:** Atieh Abedin-Do, Ze Zhang, Yvan Douville, Mirelle Méthot, Julien Bernatchez, Mahmoud Rouabhia

**Affiliations:** ^1^ Groupe de Recherche en Écologie Buccale, Faculté de Médecine Dentaire Université Laval, Quebec, QC, Canada; ^2^ Axe Médecine Régénératrice Centre de Recherche du CHU de Québec Département de Chirurgie Faculté de Médecine, Université Laval, Quebec, QC, Canada

**Keywords:** diabetes, skin equivalent, keratinocytes, electrical stimulation, foot ulcer

## Abstract

The prevalence of diabetes is increasing worldwide. Diabetes contributes to 70% of all non-traumatic lower-limb amputations, which are directly caused by diabetic foot ulcers (DFU) that are difficult to heal. Non-healing diabetic ulcers represent one of modern society’s most difficult medical challenges. One of the promising initiatives to treat DFU is the grafting of autologous skin or stimulating the skin cells at the edge of the wound to proliferate and close the wound. The present study was to engineer a diabetic human skin equivalent (DHSE) that contains fibroblasts and keratinocytes extracted from the skin collected from diabetic patients. The DHSE was used to investigate whether exposure to low-intensity electrical stimulation (ES) could promote diabetic cell activity. The ES was generated by a direct current (DC) electric field of 20 or 40 mV/mm. We demonstrated that the fibroblasts and keratinocytes could be extracted from older diabetics, cultured, and used to engineer DHSE. Interestingly, the exposure of DHSE to ES led to a structural improvement through tissue stratification, increased Ki-67 expression, and the deposition of basement membrane proteins (laminin and type IV collagen). The DHSE exposed to ES showed a high level of keratin 5 and 14 expressions in the basal and supra-basal layers. The keratinocyte proliferation was supported by an increased secretion of the keratinocyte growth factor (FGF-7). Exposure to ES decreased the activity of metalloproteinases (MMP) 2 and 9. In conclusion, we extracted keratinocytes and fibroblasts from the skin of diabetic-old donors. These cells were used to engineer skin equivalents and demonstrate that ES can promote diabetic wound healing. This DHSE can be a promising tool for various *in vitro* studies to understand the wound healing mechanisms under chronic inflammatory conditions such as diabetes. The DHSE could also be used as an autologous substrate to cover the DFU permanently.

## Introduction

Diabetes is due to losing control over blood glucose levels. Clinically, diabetic disorder includes most frequently diabetic type 1, known as insulin-dependent, and diabetic type 2, a noninsulin-dependent disease (American Diabetes Association, 2010). The World Health Organization (WHO) estimates that by 2040 diabetes will affect more than 642 million persons worldwide (International Diabetes Federation, 2017). Diabetes is considered a major epidemic of this century, responsible for a significant percentage of chronic diseases in the vascular, immune, and nervous systems (Istanbuly et al., 2022; Juraschek et al., 2022) that eventually lead to diabetic foot ulcers (DFU) (Megallaa et al., 2019). In the diabetic population, DFU is a common long-term complication with high morbidity and, in several cases, may lead to mortality. DFU is subjected to frequent infections in deep tissues leading to damage to the dermis and epidermis (Haug et al., 2022). DFU is also characterized by a prolonged inflammation phase, inadequate cytokines and growth factors, and impaired proliferation and migration of skin fibroblasts and keratinocytes (Falanga, 2005; Galkowska et al., 2006). While the pathology in diabetic ulceration is not fully understood, prolonged hyperglycemia and the consequent formation of advanced glycation end products (AGEs) are thought to play a significant role in the etiology of neuropathy and peripheral vascular diseases in diabetic patients (Tomaszewski et al., 2021). DFUs are challenging to treat and control, and as such, there is a critical need for adjunctive and efficient management protocols. Current wound care includes wound debridement, antibiotics to control infections, tissue revascularization, and off-loading plantar ulcers (Armstrong et al., 2017; Schaper et al., 2020). The delayed healing of DFU is linked to the impairment of the patient skin cells, namely fibroblasts and keratinocytes, to proliferate, migrate, differentiate, and release growth factors (Spravchikov et al., 2001; Berlanga-Acosta et al., 2020). Indeed, non-diabetic wound healing includes different well-orchestrated phases starting with the hemostasis and the activation of inflammatory cells, followed by the proliferation and migration of fibroblasts to produce the extracellular matrix (ECM) (Sorg et al., 2017). This ECM is the biological support for keratinocytes to proliferate, migrate and differentiate, forming the epidermis (Eming et al., 2014). Disrupting any wound healing phases will hamper skin wound healing, such as in DFU. Thus, the clinical management of DFU also needs to consider activating the impaired skin cells to proliferate and migrate for better DFU healing.

Promoting DFU healing could be through electrical stimulation (ES). Indeed, it has been reported that *in-vitro* exposure to ES increased the proliferation and migration of normal and diabetic human skin fibroblasts (Verdes et al., 2022). A recent study reported the acceptability and effectiveness of ES as adjunctive therapy to speed up wound healing in patients with chronic DFU and mild to severe peripheral arterial disease (Zulbaran-Rojas et al., 2021). However, the number of such studies is very limited in literature, and more studies are needed either to confirm the efficacy of ES as a tool for accelerating diabetic ulcer healing or to explore the underline mechanisms. Cutaneous wounds have been reported to generate a physiological “current of injury” that could directly participate in wound healing (Foulds et al., 1983). This is supported by the *in vitro* studies showing that ES can increase fibroblast cell proliferation and the secretion of epidermal growth factors (EGF) and fibroblast growth factors (FGF) (Wang et al., 2017).

Furthermore, a previous study showed that ES increased the production of keratin 5 (K5) and K14 by normal human skin keratinocytes (Rouabhia et al., 2020). The exposure of normal skin fibroblasts to ES modulated the production of different MMPs (Mukherjee et al., 2010). These data strongly suggest that ES may also activate diabetic human skin fibroblasts and keratinocytes to favorite cell proliferation and migration. However, ES has not been investigated in the coculture of diabetic human skin fibroblasts and keratinocytes. Such a coculture is important because of the intimate interplays between these two types of skin cells. The coculture of diabetic skin cells could be realized by engineering the diabetic human skin equivalent (DHSE). The *in vitro* reconstruction of human SE has become an important tool for investigating skin health and disease (Ali et al., 2015). SE supplies critical information about cell-cell interactions, the effects of different stimuli on epidermal and dermal cell proliferation, and differentiation. SE was also used in cosmetology testing and drug delivery testing, photoaging, and skin cancer studies (Chouinard et al., 2001; Bom et al., 2021; Graff et al., 2022). Clinically, SE was beneficial in treating three-degree burned patients (Still et al., 2003; Wisser et al., 2004; Hohlfeld et al., 2005). Thus, SE not only can be used as a platform for *in vitro* drug testing but also can play the role of a biologically active dressing where the live cells in the SE not only interact with each other but also interact with the host tissue at the edge of the ulcers, leading to cell activation, proliferation, and wound healing. Therefore, the aims of the present study were 1) to engineer a diabetic human skin tissue using the fibroblasts and keratinocytes extracted from the skin of diabetic patients and 2) to evaluate the effect of ES on the behaviors of such engineered DHSE.

## Materials and methods

### Primary diabetic human skin fibroblast and keratinocyte extraction and culture

Skin tissues were collected from diabetic patients aged between 70 and 80 years. Patients were admitted to the hospital for leg amputation due to uncontrolled foot/leg diabetic ulcers. The Université Laval-CHU Ethics Committee approved skin tissue collections. Each patient gave their informed consent before skin tissue collection. Skin tissues were cut into small pieces and treated with thermolysin (500 ug/ml) at 37°C for 24 h to separate the epidermis from the dermis. Thermolysin degrades the basal membrane zone leading to the separation of the epidermal structure from the connective tissue (Walzer et al., 1989; Gujar et al., 2021). The epidermal samples were collected and incubated with a 0.05% trypsin-0.04 ethylenediaminetetraacetic acid solution for 45 min under agitation to extract diabetic human skin keratinocyte (DHSK). The dermis samples were digested with 0.125 U/ml of collagenase-P (Boehringer Mannheim, Laval, QC, Canada) for 60 min under agitation to extract diabetic human skin fibroblast (DHSF). The fibroblasts were cultured in Dulbecco’s modified Eagle’s medium (DMEM) with 10% fetal bovine serum, 100 U/ml penicillin G, 25 μg/ml streptomycin, and 0·5 μg/ml fungizone. The keratinocytes were cultured in DMEM- Ham’s F12 (DMEMH). The keratinocyte culture medium was also supplemented with 10 ng/ml of EGF, 0.4 μg/ml of hydrocortisone, 5 μg/ml of transferrin, 5 μg/ml of insulin, 100 U/ml penicillin, 25 μg/ml streptomycin, and 0·5 μg/ml fungizone. Fibroblasts and keratinocytes were cultured in a humidified atmosphere that contained 5% CO_2_ at 37°C. The cells were then subcultured and used at passages 2 for keratinocytes and 3 for fibroblasts. The fibroblasts were identified by morphological analyses (elongated shape) and fibronectin protein expression (data not shown). The keratinocytes were identified by morphological analysis (cuboidal shape) and the expression of keratin-5 (data not shown).

### Preparation of diabetic skin equivalent

The diabetic human skin equivalent (DHSE) preparation involved two steps. The first step was to produce dermis-like tissue. For this purpose, fibroblasts (1 × 10^6^ cells) were mixed with type I bovine collagen solution (Cat. No.5005–100ML, Advanced BioMatrix, Carlsbad, US), containing 29.5% DMEM (Dulbecco’s Modified Eagle Medium) 2.7 x, 20% serum and 0.05% NaOH 0.2N. The mixture was poured into the wells of 12-well tissue culture plates containing anchorages to prevent the contraction of the engineered dermis. The plates were immediately transferred to an incubator at 37°C for 2 h to help collagen gel polymerization. At the end of this incubation period, 3 ml DMEM was added to each well and cultured in a humid atmosphere containing 5% CO_2_ at 37°C for 4 days to form the dermal equivalent. The medium was refreshed each day. The second step was to engineer the complete DHSE. Keratinocytes at 80% confluence were detached from the culture flask, counted, and seeded (9 × 10^4^/cm^2^) on the top of each dermal equivalent. These DHSE were grown under submerged conditions for 6 days to reach the keratinocyte confluence. The epidermis stratification was obtained by raising the DHSE to the air-liquid interface for 4 more days. The tissues at this stage were used for subsequent experiments.

### Preparation of conductive PPy/HE/PLLA membranes and tissue exposure to ES

Polypyrrole (PPy) particles were formed in a water-in-oil (1:3, H2O: chloroform) emulsion (1 ml) containing pyrrole (1 ml), FeCl3 (7.91 g), H2O2 (425 μl), heparin (HE) (0.5 g), and dodecylbenzenesulfonate (DBS, 1 gr). The PPy/HE particles were collected, washed with ethanol, and mixed with a PLLA solution in chloroform at a 1:9 weight ratio (PPy: PLLA). The mixture was poured into a glass Petri dish to form a thin layer and kept at room temperature in a fume hood to evaporate the chloroform, which led to the formation of the PPy/HE/PLLA membranes. These membranes were extensively washed for 24 h with deionized water, cut into the appropriate size, and inserted into a homemade electrical tissue culture device (Wang et al., 2017). The devices were then sterilized using 75% ethanol bathing for 3 × 60 min, followed by one wash with sterile PBS and then with the culture medium. A four-channel waveform generator was connected to the homemade device to supply the 20 and 40 mV/mm ES power at constant DC mode. These two ES intensities were not toxic to diabetic cells (Abidin-Do et al., 2021). The DHSE was placed on top of the membrane, with the dermis adhering to the conductive PPy/HE/PLLA membrane. The DHSEs were then subjected or not to the ES for 6 or 24 h. The time points were selected based on previous studies with normal human skin fibroblasts and keratinocytes (Park et al., 2015; Rouabhia et al., 2020). At the end of each ES period, the DHSE and the culture medium were collected and subjected to different analyses.

### Histological analysis

After exposure or not to ES, the DHSE tissues were collected, fixed in 4% paraformaldehyde, embedded in paraffin, and cut into 7 µm thick sections. The sections were treated with Bouin′s solution (HT10132, Sigma-Aldrich) and then stained with Weigert′s iron hematoxylin solution (HT107, Sigma-Aldrich) and Masson’s trichrome stain kit (Accustain HT15, Sigma-Aldrich). The tissues were observed using an epifluorescence microscope and photographed. For each tissue, three cuts were processed and observed. The experiment was done in triplicate.

### Gene expression

With a set of skin equivalents, we performed gene expression analyses. ES exposed and nonexposed tissues were lysed, and total RNA was extracted using an illustra RNAspin Mini RNA Isolation Kit (cat: 25-0500-72, GE Healthcare). The quantitative and qualitative analyses of the extracted RNA were determined by NanoDrop™ (Cat: ND-ONE-W, Thermo Scientific™). The first-strand cDNA was synthesized from 1 μg of total RNA by using a High-Capacity cDNA Reverse Transcription Kit (Cat: 4368814, Applied Biosystems, Cheshire, United Kingdom) and the T100 Thermal Cycler (1861096, Bio-Rad). The cDNA of each DHSE was used to perform Quantitative PCR (qPCR) using specific primers (Table 1) for *K5*, *K14*, *Laminin*, and *Type IV Collagen* genes. As a control, we include the GAPDH gene. The qPCR was performed in triplicate using the CFX96 Dx Real-Time PCR Detection System (cat:1841000-IVD Bio-rad). Each PCR reaction comprised 10 µl iTaq™ universal SYBR^®^ Green Supermix (cat:1725124, Bio-Rad), 2 µl cDNA (≥10 ng), 0.5 µl of each primer (5 pmol), and 6 µl of nuclease-free water in a total volume of 20 µl reaction mixture. The expression level of the GAPDH was used to normalize the test gene expression levels. The results were analyzed using the 2^−ΔΔCt^ relative expression method (Livak et al., 2001), and fold changes in the nonexposed and ES-exposed tissues were calculated and plotted.

**TABLE 1:** Real time PCR primers.

Gene	Primer sequence (5′-3′)		References	Annealing temp (^º^C)	Amplicon size (bp)
*Laminin-b3*	Forward	ATG​TGA​ACC​CTG​TCT​CTC​TGC	NM_000228.3	50	141
	Reverse	GGT​ACA​CTC​GCC​AGG​TCT​TAC			
*COL4-a2*	Forward	CTG​CCA​CTA​CTA​CGC​CAA​CA	Huang et al. (2018)	60	153
	Reverse	CCG​GCT​CAC​AGG​TTC​TTC​AT			
*K-5*	Forward	GGT​GAT​GCT​GAA​GAA​GGA​TGT​AG	NM_000424.4	60	118
	Reverse	CAG​CTC​CGC​ATC​AAA​GAA​CA			
*K-14*	Forward	CCA​GTT​CTC​CTC​TGG​ATC​GC	NM_000526.5	60	136
	Reverse	GCC​TCA​GTT​CTT​GGT​GCG​AA			
*GAPDH*	Forward	GGA​GCG​AGA​TCC​CTC​CAA​AAT	Liefke, et al. (2015)	60	197
	Reverse	GGC​TGT​TGT​CAT​ACT​TCT​CAT​GG			

### Immunohistochemistry

The immunohistochemical stains were used to evaluate the production of different proteins, including keratins 5 and 14, laminin, type IV collagen, and Ki-67. For this purpose, paraffinized DHSE tissue sections were cut on glass slides and deparaffinized. The sections were layered with one or the other of the following primary antibodies that include anti-Ki-67 (AB9260; EMD Millipore, Montréal, QC, Canada), anti-K5 (SAB5500130; Sigma-Aldrich), anti-K14 (SAB5500124; Sigma-Aldrich), anti-Laminin (ab11575; Abcam) or anti-Type IV-Collagen (C1926; Sigma-Aldrich). All antibodies were diluted in PBS 1% BSA/0.5% Tween-20. Tissues were incubated overnight at 4°C. The following day, the tissues were washed three times with PBS and incubated with secondary antibody (HRP goat anti-rabbit polyclonal secondary antibody H + L (cat:1706518. Bio-Rad) or HRP goat anti-mouse IgG H + L (cat:1706516, Bio-Rad) diluted 1/300 times in 1% BSA and 0.5% Tween-20 in PBS, and subsequently incubated for 60 min at room temperature. The tissues were washed and overlaid with diaminobenzidine tetrahydrochloride (DAB) at 1/30 dilution using the SignalStain(R) Substrate kit (8059S, Cell Signaling Technology, MA, United States) for 3 min at room temperature. The tissues were rewashed with PBS, then counterstained with hematoxylin for 3 min, dehydrated, observed under a light microscope, and photographed.

### ELISA assay

The medium of each culture was collected immediately after 6 h or 24 h exposure to ES and subjected to ELISA assays to quantify the secretion of different mediators. The Human KGF/FGF-7 Quantikine ELISA Kit (DKG00, R & D systems), Human EGF Quantikine ELISA Kit (DEG00, R & D systems), Human MMP-9 Quantikine ELISA Kit (DMP900, R & D systems) and total MMP-2 Quantikine ELISA Kit (MMP200, R & D systems) were used. The absorbance was measured at 450 nm using a Microplate Reader Model 680 (Bio-Rad, Philadelphia, PA, United States). The measurements were performed in triplicate for each condition. The minimum detectable concentrations were under 0.7 pg/ml for EGF, 15 pg/ml for FGF-7, under 30 pg/ml for MMP2, and under 160 pg/ml for MMP9, as reported by the manufacturer. Each experiment was repeated three times (*n* = 3), and the means ± SD were calculated and presented. To minimize the intra- and inter-assay variations, the collected supernatants from the different experiments (*n* = 3) were run in the same plate of either FGF-7, MMP-9, MMP-2, or EGF. Each supernatant was run in triplicate, and the value was rejected if a triplicate was over 10% different (intra-assay CV) from the other. Using the supernatants of the various experiments in the same plate gave us an inter-assay CV of less than 10%.

### Zymography

Fibroblasts and keratinocytes produce different metalloproteinases, including MMP2 and MMP9. These enzymes maintain the equilibrium of extracellular matrix synthesis and degradation. A gelatin zymography assay was performed to evaluate MMPs’ activities. The culture medium was collected from the DHSE exposed or not to 6 and 24 h ES and loaded into a 7.5% SDS-polyacrylamide gel containing 1 mg/ml of gelatin as a substrate. After electrophoresis at 125 V for 4 h, the gels were washed 2 × 30 min with 2.5% Triton X-100 at room temperature. The gels were then incubated 16 h at 37°C in an activation buffer (0.05 M Tris–HCl, 0.05 M NaCl, 10 mM CaCl_2_, 0.05% Brij 35, pH 7.6). They were stained with 0.5% Coomassie Blue R-250 for 60 min and destained twice using a 40% methanol-10% acetic acid solution. The gels were photographed and analyzed with ImageJ 1.47v software. The experiment was repeated three separate times.

### Statistical analysis

One-way ANOVA was used to determine the statistical significance of the differences between the control and test values. Posteriori comparisons were performed using Tukey’s method. Normality and variance assumptions were verified using the Shapiro–Wilk test and the Brown and Forsythe test, respectively. Differences were declared significant when *p*-values were found ≤0.05. Data were analyzed using the GraphPad INSTAT software.

## Results

### Diabetic tissue structure with and without exposure to ES

**FIGURE 1:**
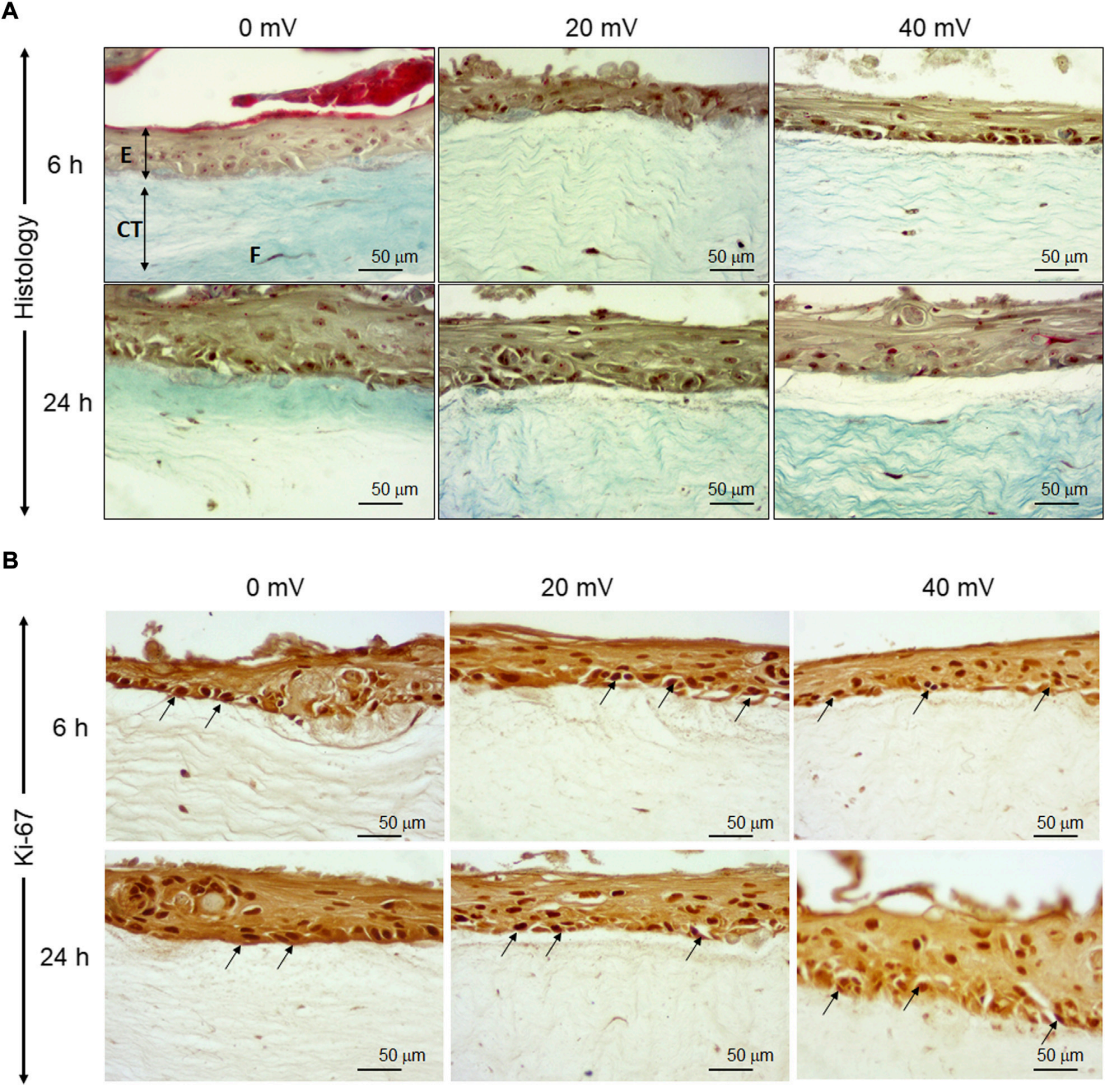
Tissue structure of diabetic skin equivalent after exposure or not to electrical stimulation. Skin equivalents were generated using fibroblasts and keratinocytes extracted from the skin of diabetic donors. The generated skin equivalents were exposed or not to electrical stimulation at 20 or 40 mV/mm. Immediately after exposure to ES, the tissues were subjected to structural analyses. Panel A shows Masson trichrome-stained tissues. Panel B shows Ki-67-stained cells. (E), epidermis; (CT), connective tissue; (F), Fibroblasts.

As shown in Figure 1A, the epidermis comprises several layers of cells in the control groups (0 mV/mm). In the dermis, fibroblasts are oriented in parallel with the collagen fibers. In the test groups, the exposure of the DHSE to 20 mV/mm and 40 mV/mm for 6 and 24 h has no macroscopic effect on tissue structures. The epidermis forms several well-structured layers on the dermis. Altogether, these results demonstrated the feasibility of generating skin equivalents using the fibroblasts and keratinocytes extracted from the human skin of diabetic donors. To further characterize the engineered DHSE, we performed Ki-67 staining as a proliferating cell marker. Figure 1B shows a high number of Ki-67 positive cells in control (0 mV/mm) tissues confirming the capacity of the diabetic keratinocyte to proliferate. Interestingly, the keratinocytes exposed to 20 and 40 mV/mm show a higher Ki-67 cell density than the control (nonexposed cells). Overall, our study is the first to demonstrate the possibility of engineering a SE that contains a fibroblast-populated collagen matrix and a stratified epidermis generated with the keratinocytes and fibroblasts extracted from the skin of diabetic donors. The engineered DHSEs in this experiment were obtained using the fibroblasts and keratinocytes extracted from the same donors.

### The expression and production of basement membrane by the DHSE

**FIGURE 2:**
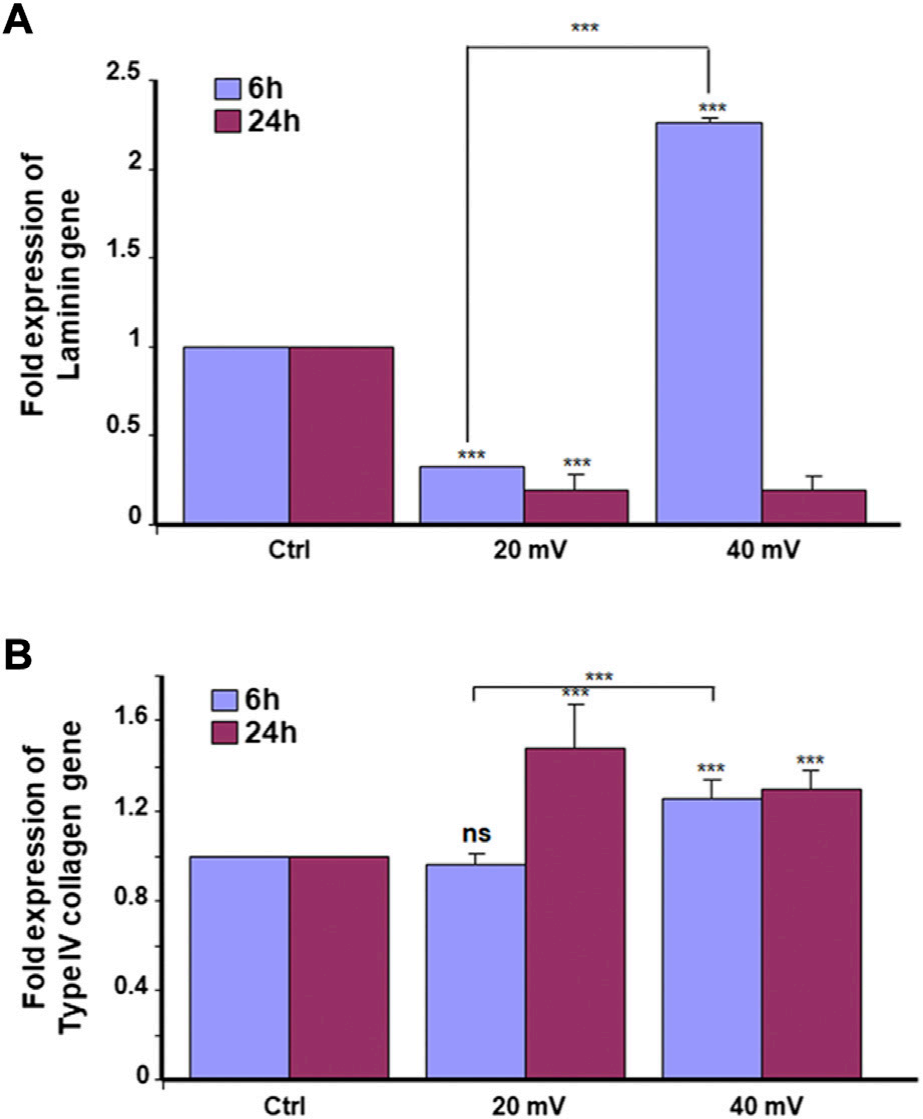
Laminin **(A)** and type IV collagen **(B)** expressions. The tissues were exposed for 6 and 24 h to ES. Total RNA was extracted from the tissues following each culture period and was subjected to quantitative RT-PCR of the laminin and Type IV collagen genes using specific premiers. Results are presented as a fold expression of the gene in the test sample compared to this gene expression in control. Data are expressed as means ± SD from triplicate assays of three different experiments. (ns), non-significant. ***p ˂ 0.001. Free asterisks refer to the statistical difference when comparing the ES-exposed and nonexposed (Ctrl) tissues. Bars with asterisks show the comparison of the different intensities of ES.

**FIGURE 3:**
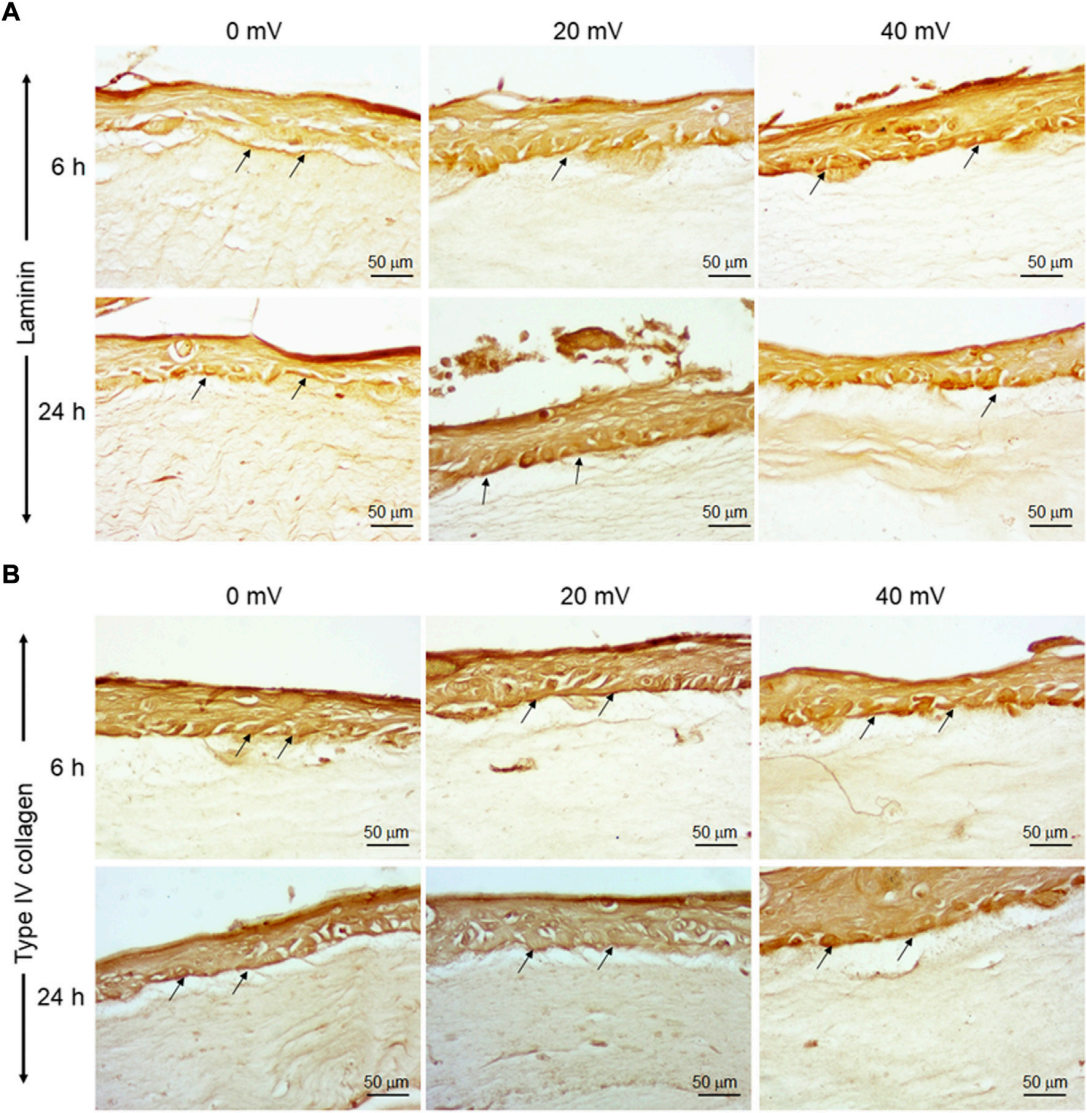
Deposition of laminin and Type IV collagen at the basement membrane of DHSE. After the tissue engineering and exposure to ES for 6 and 24 h, the presence of laminin and Type IV collagen proteins were evaluated by immunohistochemistry, as described in the M&M. Panel A shows Laminin-stained tissues. Panel B shows Type IV collagen-stained tissues. Arrows point out stained proteins. (*n* = 4).

The keratinocytes and fibroblasts cooperated in DHSE synthesized dermal-epidermal proteins. At the gene expression level, our results showed (Figure 2A) that the 6 h exposure to 20 mV/mm decreased laminin expression, but the same period of exposure to 40 mV/mm increased laminin expression. While for the 24 h exposure, laminin gene expression decreased at 20 and 40 mV/mm. However, the type IV collagen gene expression was increased after 24 h exposure to either 20 or 40 mV/mm. On the other hand, for the 6 h exposure, only the 40 mV/mm intensity led to a significant (*p* < 0.001) increase in type IV collagen gene expression (Figure 2B). These results with gene expression were supported by the immunohistochemical stains of laminin and type IV collagen proteins. As shown in Figure 3A, both nonexposed and ES-exposed tissues secreted laminin protein (arrows). Similar results were obtained for type IV collagen protein. Figure 3B showed (arrows) collagen type IV deposition between the epidermis and the dermis.

### K5 and K14 expressions and productions by DHSE

**FIGURE 4:**
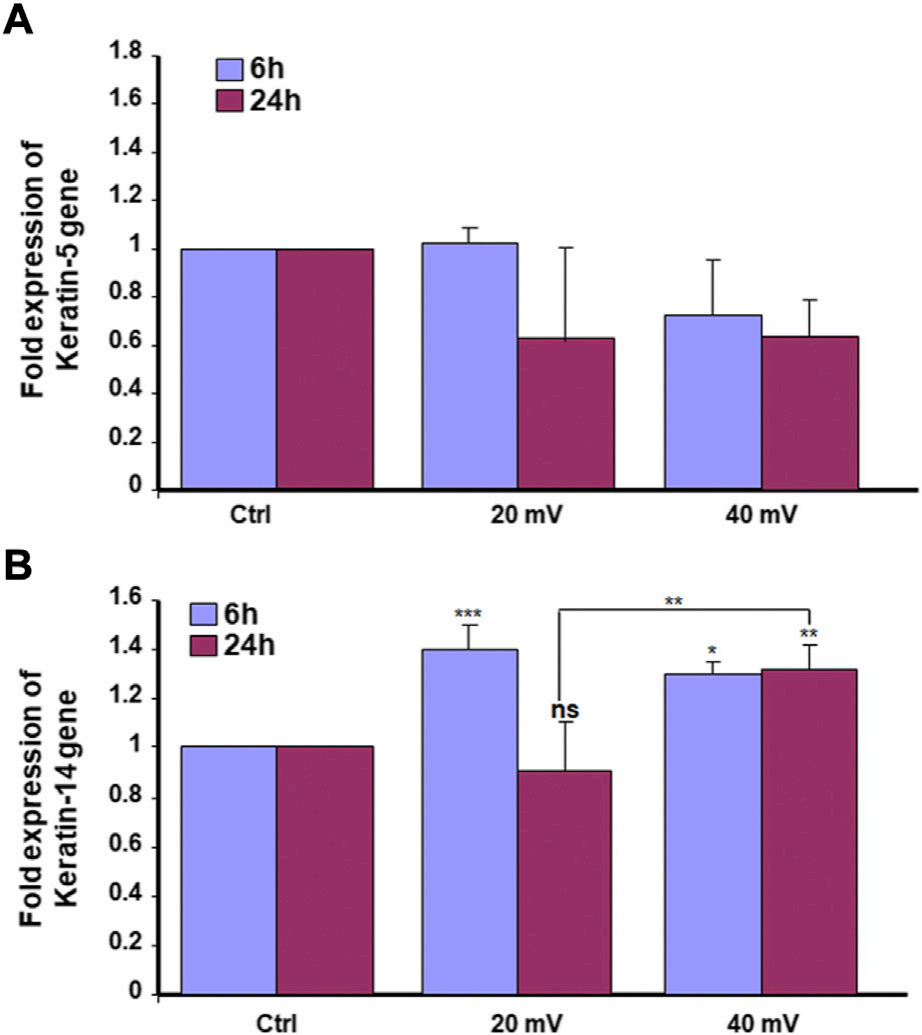
Exposure to ES increased the expression of K5 **(A)** and K14 **(B)** genes. The DHSEs were generated and exposed for 6 and 24 h to 20 mV/mm or 40 mV/mm. The tissues were used to extract RNA and perform qRT-PCR to evaluate the expression levels of K5 and K14. Results are presented as a fold expression of the gene in the test sample compared to this gene expression in control. Data are expressed as means ± SD from triplicate assays of three different experiments. (ns), non-significant. *p ˂ 0.05, **p ˂ 0.01. Free asterisks refer to the statistical difference when comparing the ES-exposed and nonexposed (Ctrl) tissues. Bars with asterisks show the comparison of the different intensities of ES.

**FIGURE 5:**
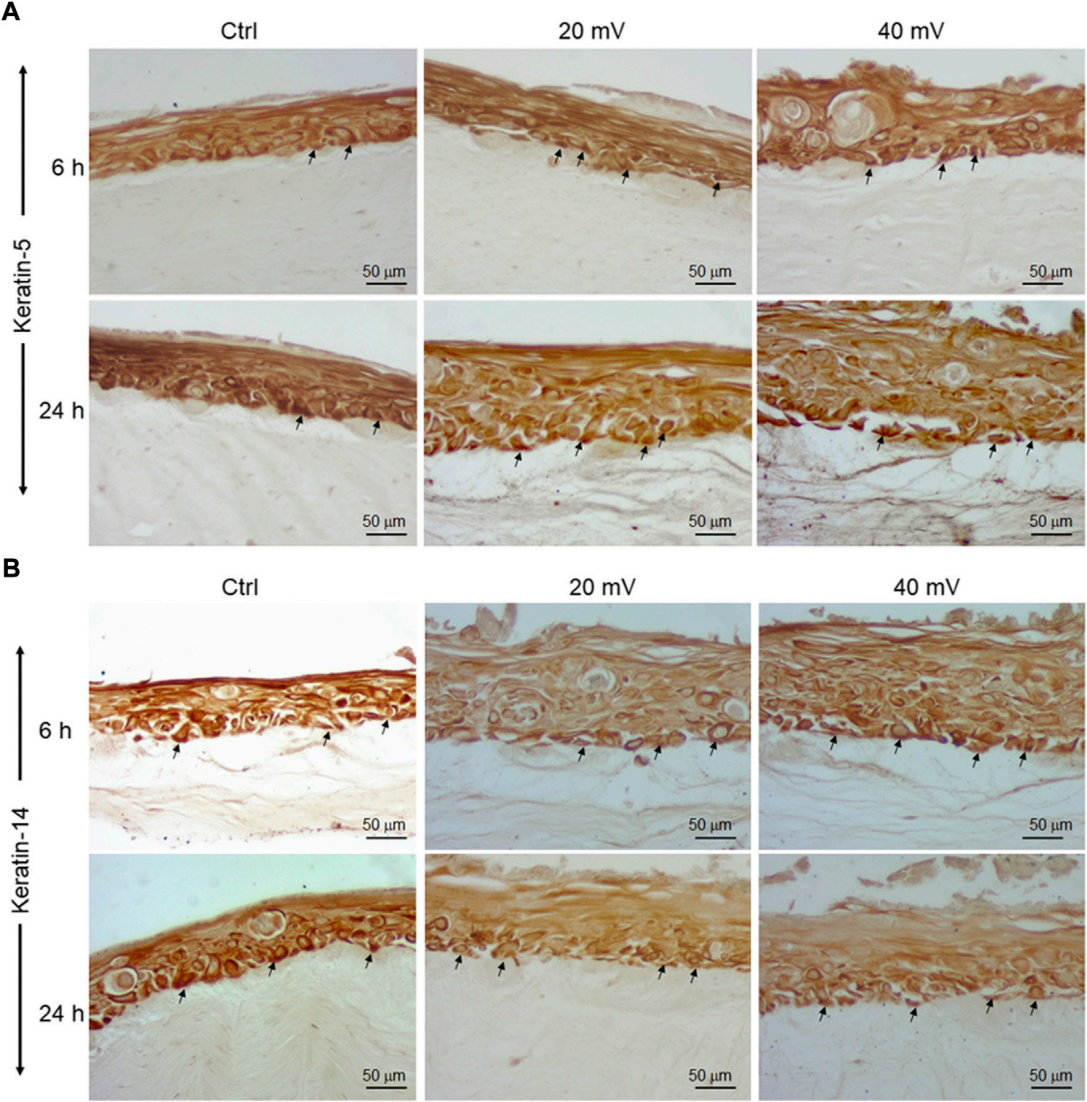
K5 **(A)** and K14 **(B)** positive cells in DHSE after exposure or not to ES. The DHSEs were exposed for 6 and 24 h to 20 mV/mm or 40 mV/mm. Tissue sections were stained with specific antibodies to K5 and K14, respectively. Photos are representative of 3 different experiments, with each condition performed in triplicate. Arrows showed the stained cells at the basal and suprabasal layers.

Proliferating keratinocytes express different types of proteins at the basal and supra-basal layers. In this study, we analyzed the expression of K5 (Figure 4A) and K14 (Figure 4B) genes by the DHSE. As shown in gene expressions were increased. Specifically, the exposure to ES for 6 h led to a significant increase of K14 at both 20 and 40 mV/mm. The exposure for 24 h to ES led to a significantly increased expression of K14 at 40 mV/mm, only. The protein analyses supported the gene expression analyses. As shown in Figure 5, there are several K5 and 14 positive cells after immunohistochemical staining. Figure 5A shows the K5 positive cells at the basal and supra-basal layers (arrows). The density of the K5 positive cells appears similar when comparing the ES-exposed to nonexposed tissues, confirming the gene expression analyses (Figure 4). Similar observations apply to the K14 (Figure 5B).

### Secretion of EGF and FGF by DHSE

**FIGURE 6:**
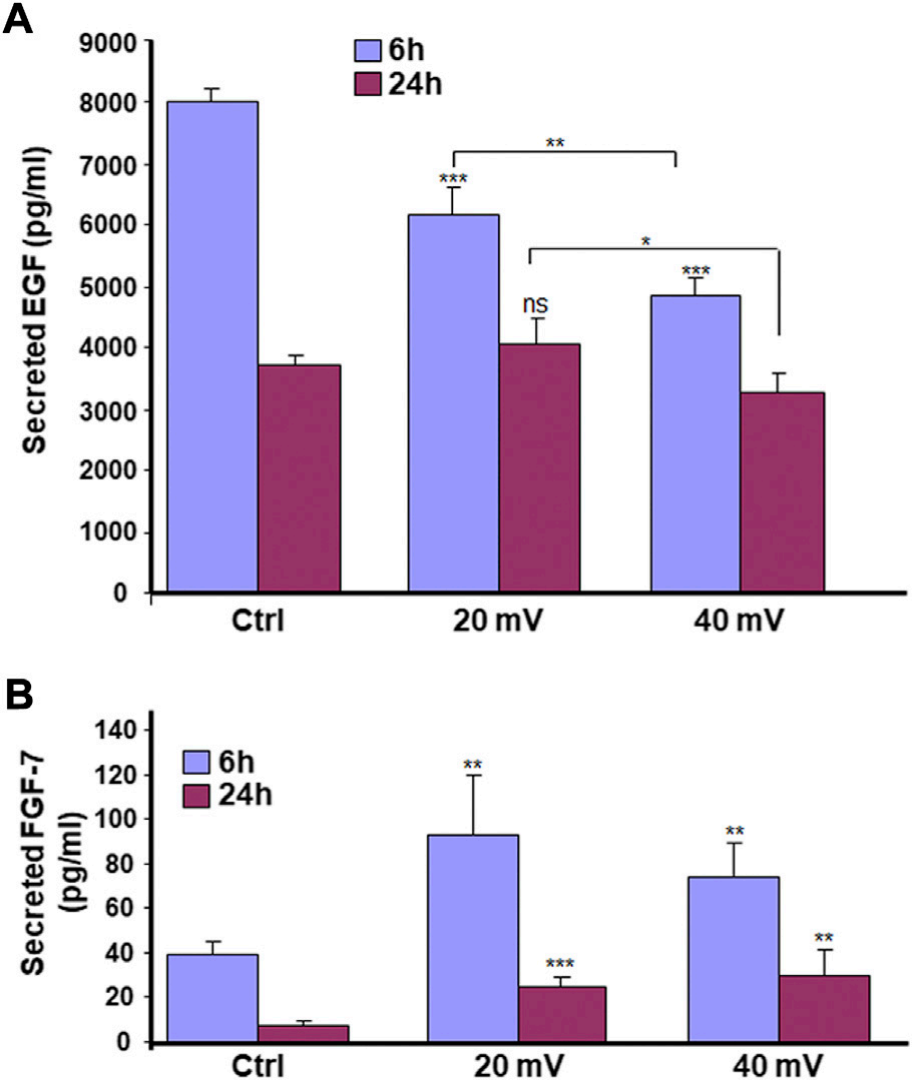
ES modulates the secretion of EGF **(A)** and FGF-7 **(B)** by diabetic skin cells. DHSE were exposed to 20 or 40 mV/mm of ES for 6 and 24 h. Immediately after each exposure period, the culture medium was used to measure le levels of EGF and FGF-7 as reported in the M&M. Results are expressed as means ± SD from triplicate assays of three different experiments. (ns), non-significant. *p ˂ 0.05, **p ˂ 0.01, ***p ˂ 0.001. Free asterisks refer to the statistical difference when comparing the ES-exposed and nonexposed (Ctrl) tissues. Bars with asterisks show the comparison of the different intensities of ES.

As shown in Figure 6A, the EGF secretions decreased over time for both control and test groups. It was the highest in the control group at 6 h but became similar for all three groups at 24 h. The exposure to 20 and 40 mV/mm for 6 h may have accelerated the decrease of the EGF. Furthermore, the reduction observed at 6 h was more significant at 40 mV/mm compared to 20 mV/mm. The measurement of FGF-7 secretion showed (Figure 6B) a significant increase for both the 6 and 24 h exposures at both 20 mV/mm and 40 mV/mm.

### Production of MMP-2 and MMP-9 by the DHSE

**FIGURE 7:**
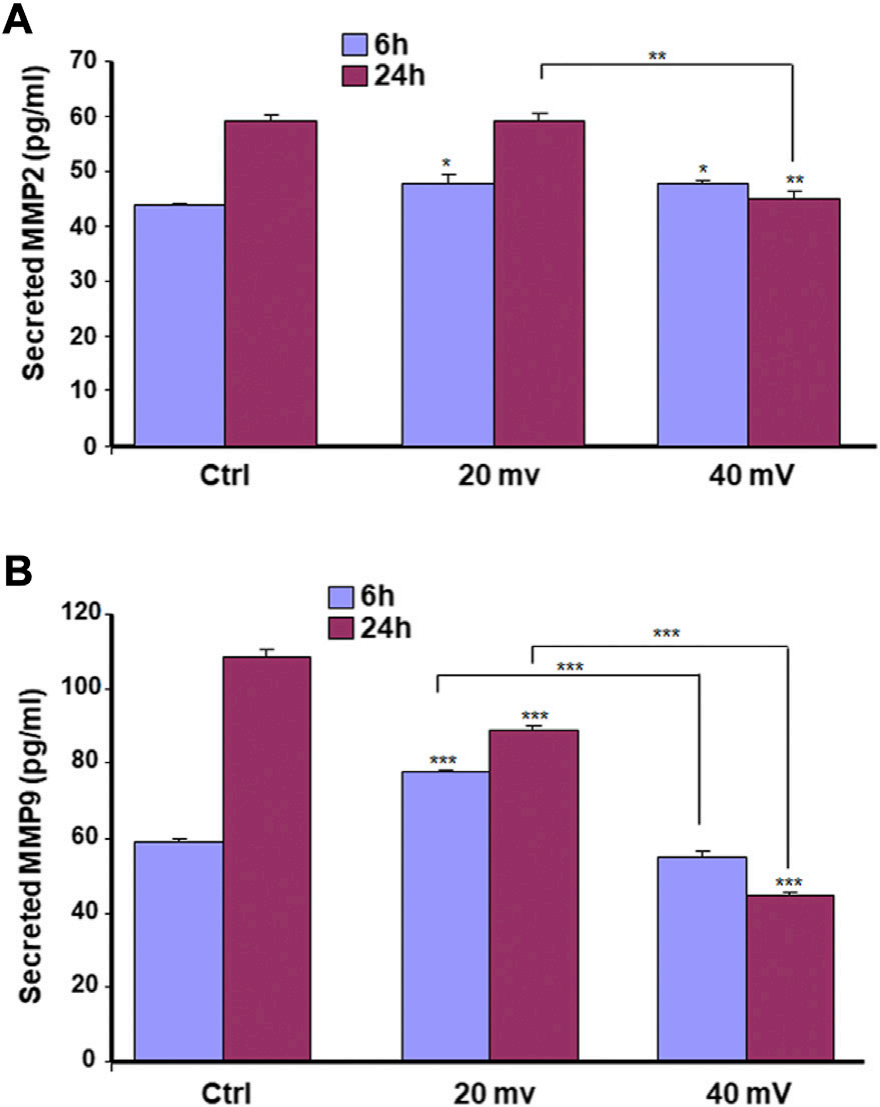
ES modulates the secretion of MMP2 **(A)** and MMP9 **(B)** by diabetic skin cells. DHSE were exposed to 20 or 40 mV/mm of ES for 6 and 24 h. Immediately after each exposure period, the culture medium was used to measure le levels of MMP2 and MMP9 as reported in the M&M. Results are expressed as means ± SD from triplicate assays of three different experiments. (ns), non-significant. *p ˂ 0.05, **p ˂ 0.01, ***p ˂ 0.001. Free asterisks refer to the statistical difference when comparing the ES-exposed and nonexposed (Ctrl) tissues. Bars with asterisks show the comparison of the different intensities of ES.

**FIGURE 8:**
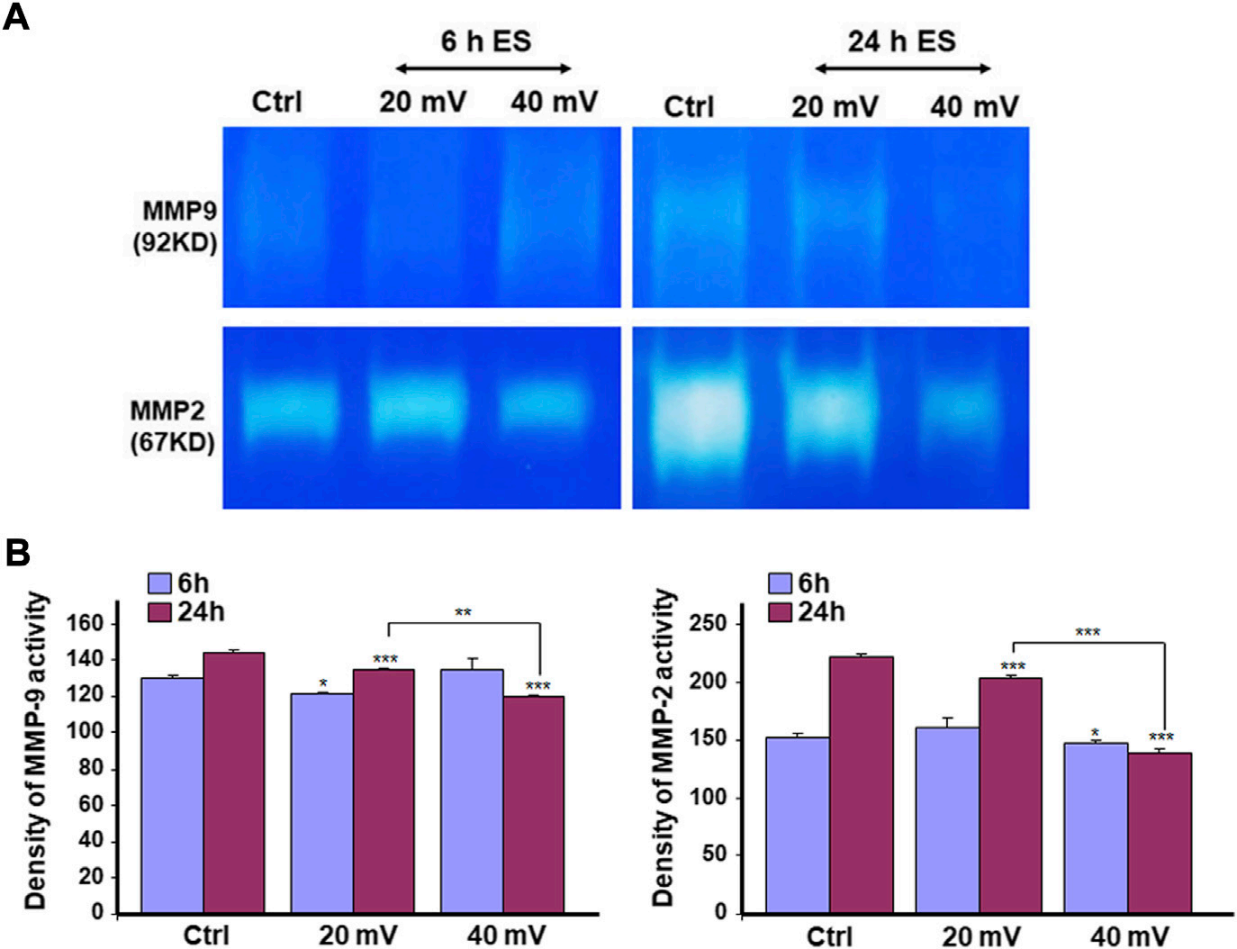
ES decreased MMP2 **(A)** and MMP9 **(B)** proteolytic activities. DHSE were exposed to 20 or 40 mV/mm of ES for 6 and 24 h. Immediately after each exposure period, the culture medium was used to evaluate the gelatin digestion using a zymography assay, as reported in the M&M. Representative gels from three experiments were photographed and presented. Quantitative measurement was performed using ImageJ analysis. *p ˂ 0.05, **p ˂ 0.01 when comparing the ES-exposed and nonexposed conditions.

We investigated the production of MMP-2 and MMP-9 by the DHSE, showing (Figure 7) that cells in the engineered DHSE could produce MMP-2 and MMP-9 at detectable levels in both control and test groups. For the MMP-2, this enzyme was slightly increased after the 6 h exposure to 20 and 40 mV/mm (Figure 7A). However, after 24 h exposure to ES, the MMP-2 levels were unchanged at 20 mV/mm, but decreased at 40 mV/mm. For the MMP-9, both ES intensities (20 mV/mm and 40 mV/mm) led to a significant (*p* < 0.001) decrease except the 6 h ES at 20 mV/mm that increased MMP-9 (*p* < 0.001) (Figure 7B). We performed a zymography assay to evaluate the proteolytic activities of these MMP-2 and MMP-9 enzymes. As shown in Figure 8A, moderate gelatin degradation bands were referring to the MMP-9 activity. The degradation was higher in control than with the ES-exposed tissues after the 24 h exposure to ES. On the other hand, 24 h ES significantly decreased the MMP-2 activity towards gelatin, as the lysis band is stronger for the control than the ES groups. This means that the exposure for 24 h to ES led to a higher MMP2 activity inhibition (Figure 8A). Semi-quantitative analysis confirmed such a decrease in MMP-2 and MMP-9 activities against gelatin (Figure 8B). Overall, the cells in the engineered DHSE showed MMP-2 and MMP-9 activities, which were decreased by exposure to ES.

## Discussion

Chronic diseases such as diabetes can have important adverse effects on patient health and well-being. These adverse effects may include skin disorders such as improper wound healing (Eming et al., 2014). DFU is an example of the consequence of chronic wounds that can lead to significant morbidity, mortality, hospitalization, and limb amputation (Brownrigg et al., 2013; Chen et al., 2017; Liu et al., 2020). Although there are several therapeutic initiatives to treat DFU, the efficiency of these initiatives is still to be improved. The most appropriate treatment for DFU must be able to promote the proliferation of the patient skin cells, increase the extracellular matrix production to fill up the wounded area, and regulate tissue angiogenesis. The two major cells involved in wound healing are fibroblasts and keratinocytes (Amiri et al., 2022). For diabetic patients, the proliferative capacity of these cells is highly reduced due to the wound site’s persistent inflammation (Lerman et al., 2003; Rizwan et al., 2020). To change such a situation, it is possible to extract keratinocytes and fibroblasts from the skin of diabetic donors and grow them *in vitro* for both *in vitro* and/or *in vivo* uses. In this study, we first reported the possibility of extracting and subculturing keratinocytes and fibroblasts from the skin of diabetic patients. We noticed that the keratinocytes and fibroblasts have a limited capacity for subculturing. Indeed, starting from passage 3, the keratinocytes showed differentiating phenotypes. They showed a significant morphological change in becoming large-sized cells, stopping growing and detaching from the cell culture plates. A similar observation was found with the diabetic fibroblasts starting at passage 6. The reduced capacity of keratinocytes and fibroblasts to proliferate could be due to diabetic conditions (Lerman et al., 2003; Rizwan et al., 2020) and donor age, as previously reported (Parkinson et al., 1987; Ding et al., 2021). Considering these conditions (diabetes and donor age), we used diabetic skin cells at low passages to engineer the DHSE. The engineered tissue was well-structured, showing a fibroblast-populated connective tissue and a multilayered epidermis. Such an engineered DHSE could be used as an autologous skin graft, as it is generated with the patient cells to avoid rejection. The engineered DHSE could also be used for *in vitro* studies to evaluate potential clinical applications such as ES and drug testing. Indeed, we have shown for the first time that ES improved *in vitro* the diabetic tissue structure and basement membrane protein production. These results are similar to those previously reported with normal human skin cells (Rouabhia et al., 2016). They suggest using ES to promote growth and tissue formation in diabetic patients. The engineered DHSE demonstrated that ES improved the DHSE structure by promoting the production of laminin and type IV collagen. Laminin and type IV collagen are the key proteins found in the basement membrane. They contribute to the epidermis interaction with the dermis (Watt, 2014). The presence of laminin and type IV collagen in the ES-exposed DHSE confirmed the efficacy of ES in promoting the growth of both fibroblasts and keratinocytes and their interactions through the basement membrane. ES also promoted the expression of the cell proliferating marker, Ki-67. The expression of Ki-67 is associated with healthy cells contributing to tissue formation (Shankar et al., 2020). That the ES promoted the Ki-67 production in the DHSE confirmed the positive effect of ES in promoting diabetic skin cell proliferation. This was confirmed by the increased K14 expression and production by the diabetic keratinocytes. Ki-67 and K14 are important to epidermal structure and functions (Raja-Sivamani et al., 2007). Proliferating keratinocytes produce keratins, a family of fibrous structural proteins providing structural stability to the skin epidermis (Knöbel et al., 2015). Keratin 5/keratin 14 interaction is vital to the integrity of basal keratinocytes and the development and maintenance of epidermal structures (Wagner et al., 2012). These keratins (K5 and K14) can be modulated when epithelial cells are subjected to stimulating or inhibiting wound healing conditions. Our findings show that ES promoted keratin expression in DHSE, suggesting a possible beneficial effect of ES by stimulating the production of keratins by diabetic keratinocytes and ultimately promoting wound healing. Further research will shed more light on the mechanisms underlining the diabetic cell activation by ES to produce K14. The ES promoted the expression of proliferating markers such as Ki-67, and the production of epidermal structural proteins K5 and K14 could have been supported by the increased secretion of FGF-7, as demonstrated in this study. FGF-7 is an important contributing factor in cell growth, as it stimulates the growth and differentiation of different cell types, including fibroblasts and keratinocytes (Jettanacheawchankit et al., 2009). Thus, the upregulated FGF-7 production confirmed the usefulness of ES in promoting the proliferation of diabetic human skin cells contributing to wound healing. Regulating wound healing involves different proteolytic enzymes, including MMP-2 and MMP-9. Both enzymes contribute to the equilibrium of extracellular matrix production and degradation (Dissemond et al., 2020). With the present study, we demonstrated that cells in the engineered DHSE produced a detectable amount of MM-2 and MMP-9. Interestingly, the levels and activities of these enzymes were decreased following the exposure of the tissues to ES. The decrease of MMP-2 and MMP-9 suggests an accelerated buildup of ECM for better tissue regeneration. In other words, ES could promote wound healing by decreasing proteolytic enzyme activities to reduce the degradation of the needed ECM. This work, therefore, highlights the potential of using the DHSE, possibly in conjunction with ES, for therapeutic and analytic applications. It also warrants *in vivo* experiments to examine such potential.

Conclusion. We reported the possibility of extracting and culturing keratinocytes and fibroblasts from diabetic human skin. The cells were successfully used for the first time to engineer a DHSE. ES was found effective in improving the skin tissue structure through the proliferation of cells and the production of basement membrane proteins. The ES also promoted the secretion of FGF-7 but not EGF. The levels and activities of MMP-2 and MMP-9 were decreased. Altogether, these results demonstrated the possibility of engineering an autologous DHSE and its use for *in vitro* and *in vivo* applications.

## Data Availability

All datasets generated for this study are included in the article/supplementary material.
